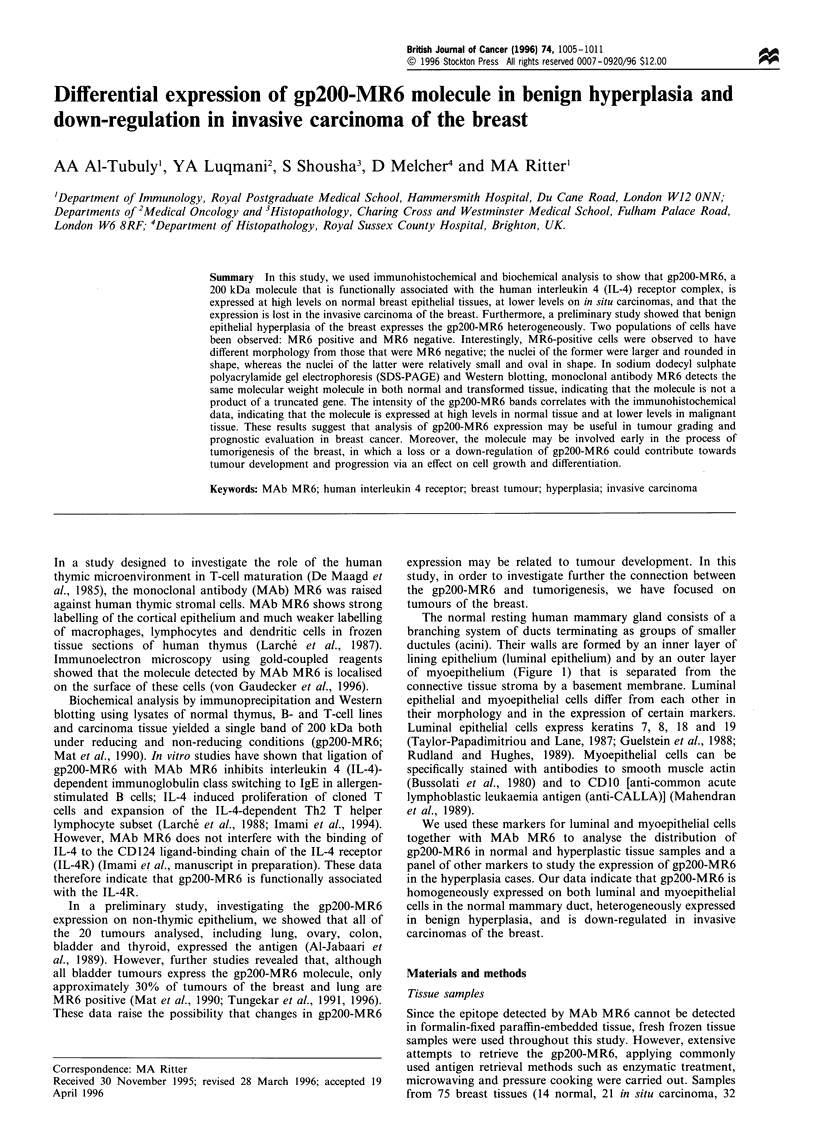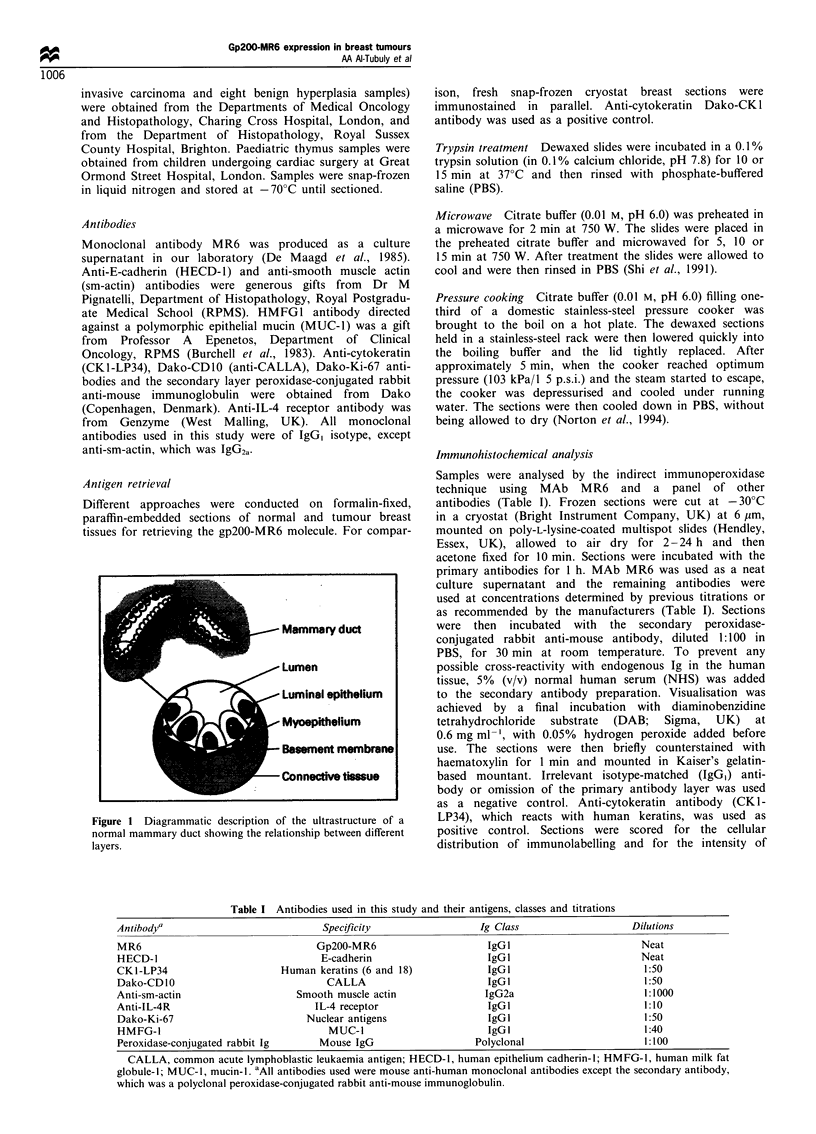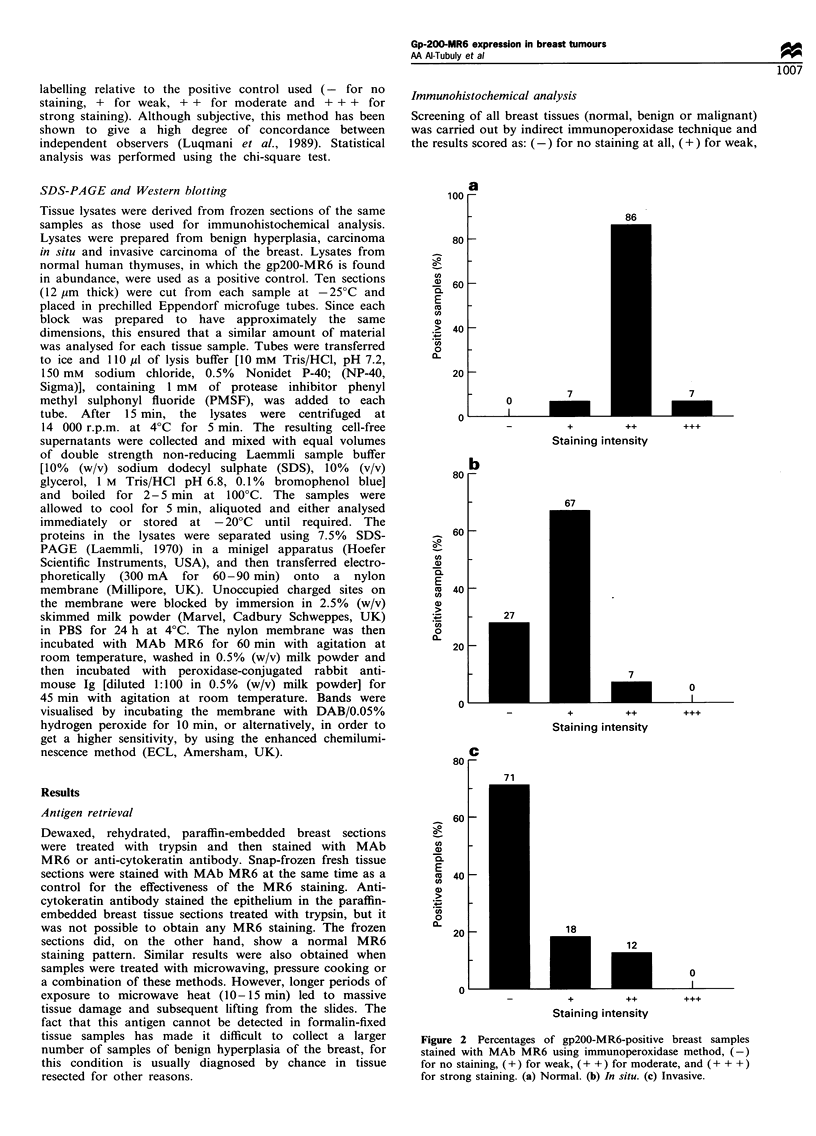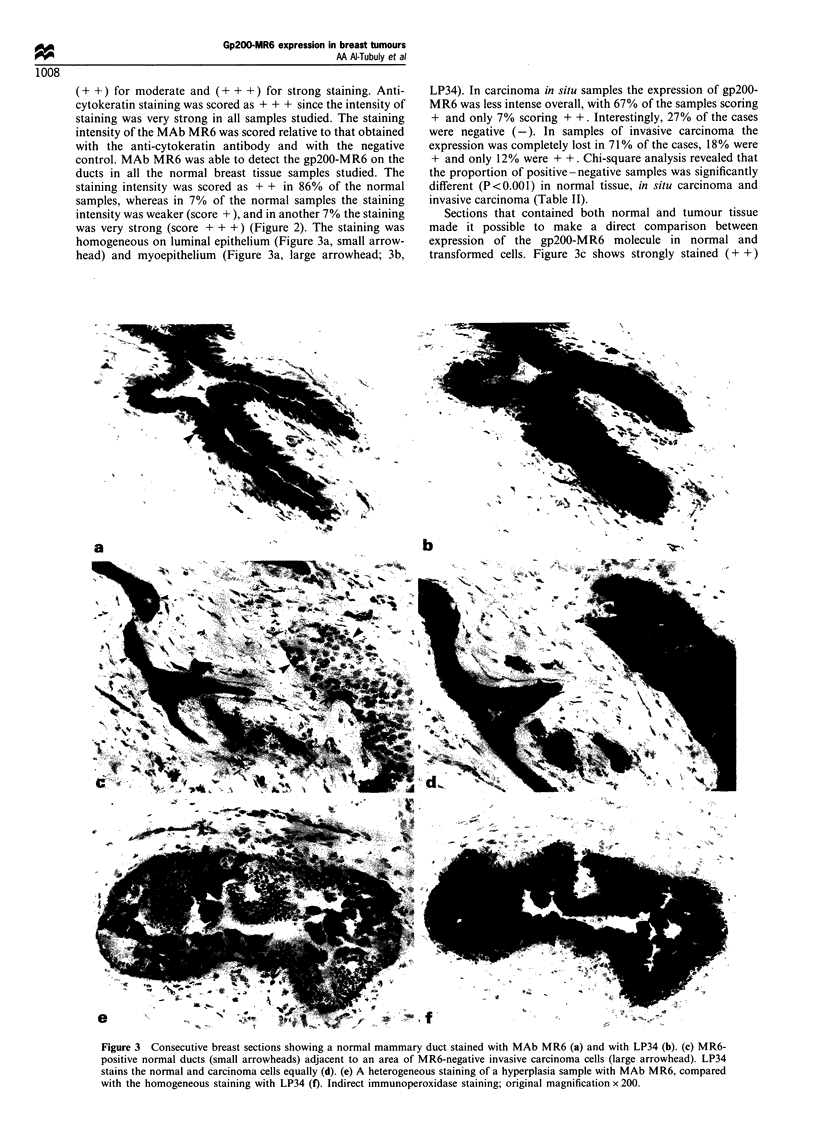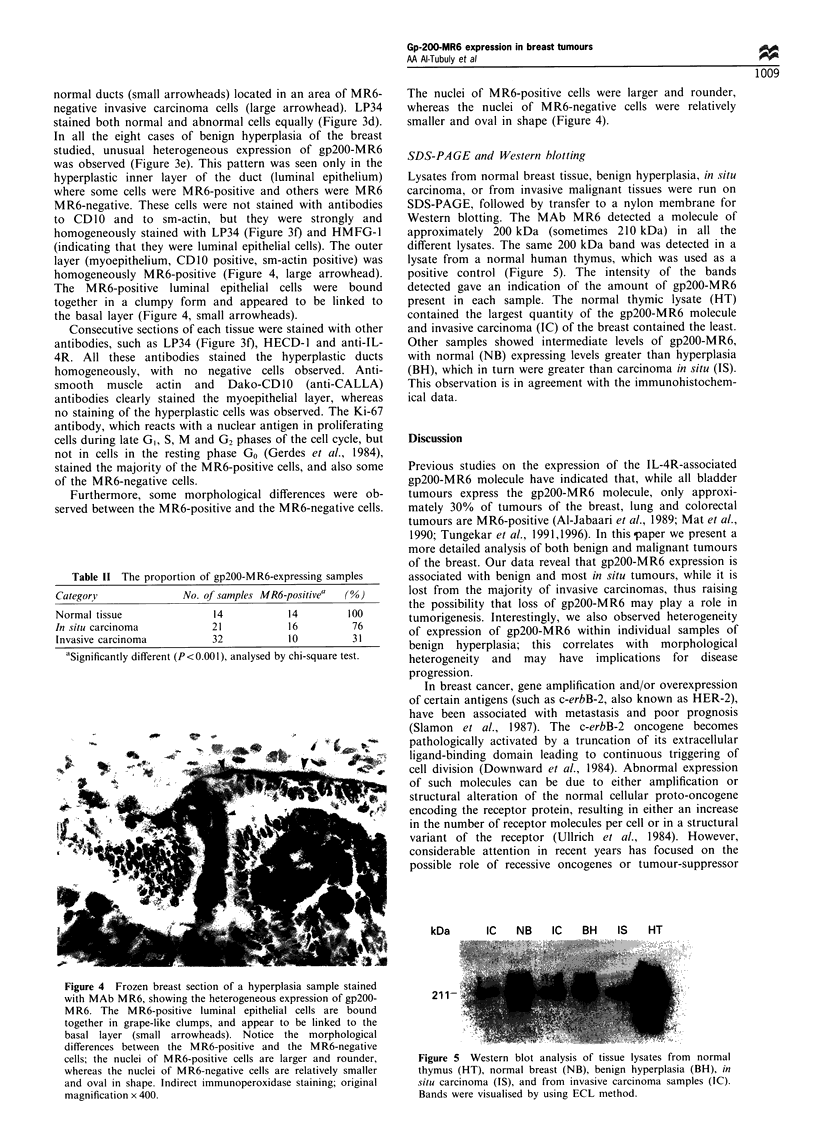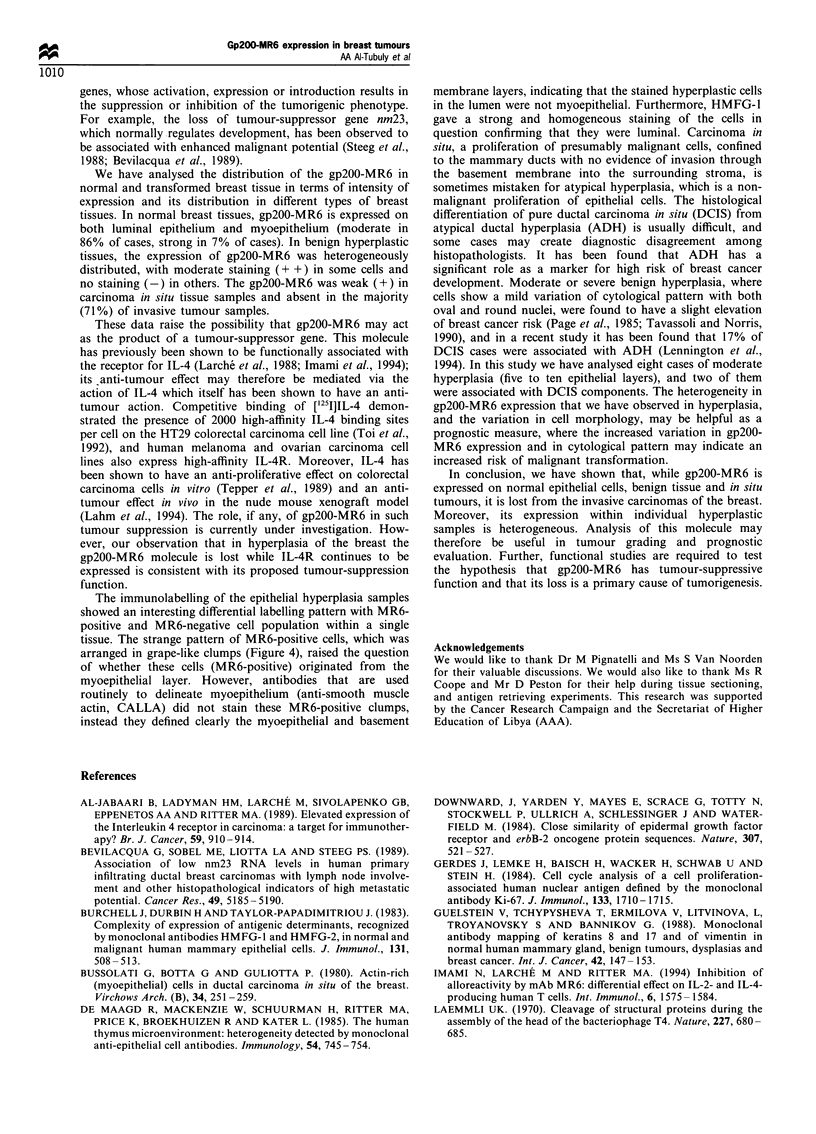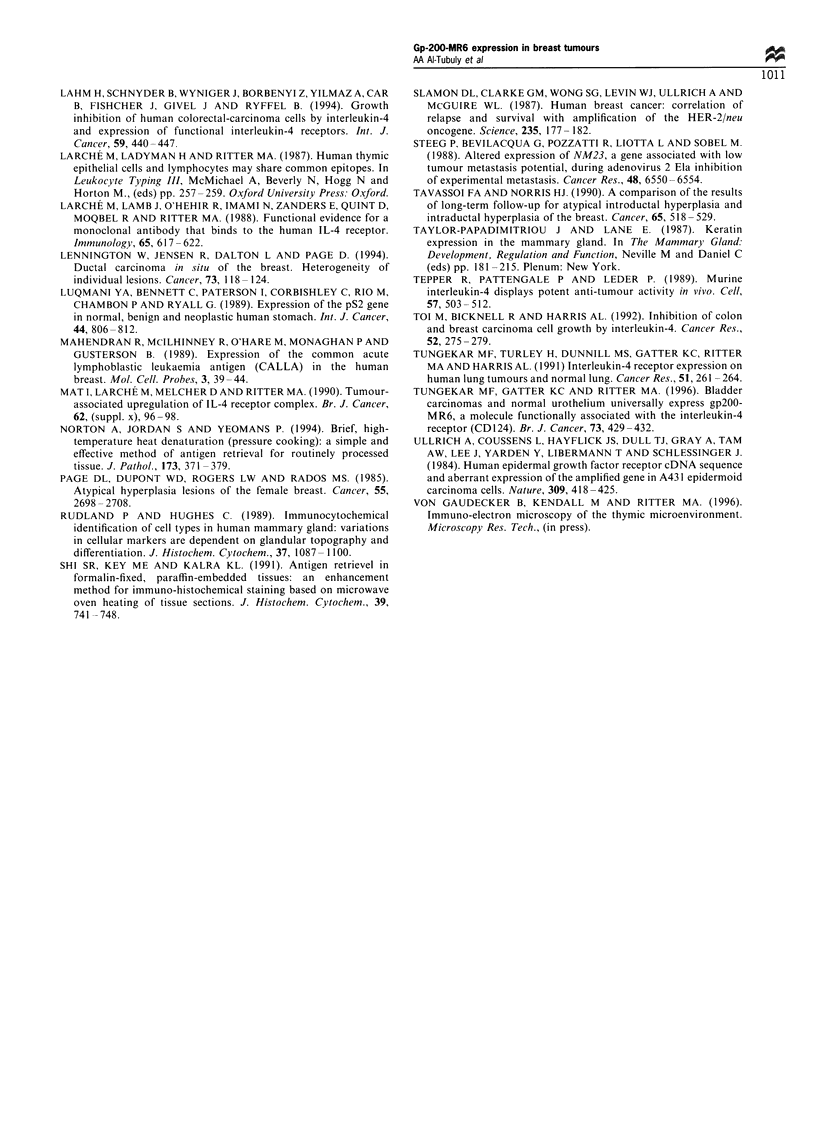# Differential expression of gp200-MR6 molecule in benign hyperplasia and down-regulation in invasive carcinoma of the breast.

**DOI:** 10.1038/bjc.1996.481

**Published:** 1996-10

**Authors:** A. A. al-Tubuly, Y. A. Luqmani, S. Shousha, D. Melcher, M. A. Ritter

**Affiliations:** Department of Immunology, Royal Postgraduate Medical School, Hammersmith Hospital, London, UK.

## Abstract

**Images:**


British Journal of Cancer (1996) 74, 1005-1011
© 1996 Stockton Press All rights reserved 0007-0920/96 $12.00

### Differential expression of gp200-MR6 molecule in benign hyperplasia and down-regulation in invasive carcinoma of the breast

AA Al-Tubuly[1], YA Luqmani[2], S Shousha[3], D Melcher[4] and MA Ritter[1]

[1]Department of Immunology, Royal Postgraduate Medical School, Hammersmith Hospital, Du Cane Road, London W12 0NN; Departments of [2]Medical Oncology and [3]Histopathology, Charing Cross and Westminster Medical School, Fulham Palace Road, London W6 8RF; [4]Department of Histopathology, Royal Sussex County Hospital, Brighton, UK.

**Summary** In this study, we used immunohistochemical and biochemical analysis to show that gp200-MR6, a 200 kDa molecule that is functionally associated with the human interleukin 4 (IL-4) receptor complex, is expressed at high levels on normal breast epithelial tissues, at lower levels on *in situ* carcinomas, and that the expression is lost in the invasive carcinoma of the breast. Furthermore, a preliminary study showed that benign epithelial hyperplasia of the breast expresses the gp200-MR6 heterogeneously. Two populations of cells have been observed: MR6 positive and MR6 negative. Interestingly, MR6-positive cells were observed to have different morphology from those that were MR6 negative; the nuclei of the former were larger and rounded in shape, whereas the nuclei of the latter were relatively small and oval in shape. In sodium dodecyl sulphate polyacrylamide gel electrophoresis (SDS-PAGE) and Western blotting, monoclonal antibody MR6 detects the same molecular weight molecule in both normal and transformed tissue, indicating that the molecule is not a product of a truncated gene. The intensity of the gp200-MR6 bands correlates with the immunohistochemical data, indicating that the molecule is expressed at high levels in normal tissue and at lower levels in malignant tissue. These results suggest that analysis of gp200-MR6 expression may be useful in tumour grading and prognostic evaluation in breast cancer. Moreover, the molecule may be involved early in the process of tumorigenesis of the breast, in which a loss or a down-regulation of gp200-MR6 could contribute towards tumour development and progression via an effect on cell growth and differentiation.

**Keywords:** MAb MR6; human interleukin 4 receptor; breast tumour; hyperplasia; invasive carcinoma

In a study designed to investigate the role of the human thymic microenvironment in T-cell maturation (De Maagd *et al*., 1985), the monoclonal antibody (MAb) MR6 was raised against human thymic stromal cells. MAb MR6 shows strong labelling of the cortical epithelium and much weaker labelling of macrophages, lymphocytes and dendritic cells in frozen tissue sections of human thymus (Larché *et al*., 1987). Immunoelectron microscopy using gold-coupled reagents showed that the molecule detected by MAb MR6 is localised on the surface of these cells (von Gaudecker *et al*., 1996).

Biochemical analysis by immunoprecipitation and Western blotting using lysates of normal thymus, B- and T-cell lines and carcinoma tissue yielded a single band of 200 kDa both under reducing and non-reducing conditions (gp200-MR6; Mat *et al*., 1990). *In vitro* studies have shown that ligation of gp200-MR6 with MAb MR6 inhibits interleukin 4 (IL-4)-dependent immunoglobulin class switching to IgE in allergen-stimulated B cells; IL-4 induced proliferation of cloned T cells and expansion of the IL-4-dependent Th2 T helper lymphocyte subset (Larché *et al*., 1988; Imami *et al*., 1994). However, MAb MR6 does not interfere with the binding of IL-4 to the CD124 ligand-binding chain of the IL-4 receptor (IL-4R) (Imami *et al*., manuscript in preparation). These data therefore indicate that gp200-MR6 is functionally associated with the IL-4R.

In a preliminary study, investigating the gp200-MR6 expression on non-thymic epithelium, we showed that all of the 20 tumours analysed, including lung, ovary, colon, bladder and thyroid, expressed the antigen (Al-Jabaari *et al*., 1989). However, further studies revealed that, although all bladder tumours express the gp200-MR6 molecule, only approximately 30% of tumours of the breast and lung are MR6 positive (Mat *et al*., 1990; Tungekar *et al*., 1991, 1996). These data raise the possibility that changes in gp200-MR6 expression may be related to tumour development. In this study, in order to investigate further the connection between the gp200-MR6 and tumorigenesis, we have focused on tumours of the breast.

The normal resting human mammary gland consists of a branching system of ducts terminating as groups of smaller ductules (acini). Their walls are formed by an inner layer of lining epithelium (luminal epithelium) and by an outer layer of myoepithelium (Figure 1) that is separated from the connective tissue stroma by a basement membrane. Luminal epithelial and myoepithelial cells differ from each other in their morphology and in the expression of certain markers. Luminal epithelial cells express keratins 7, 8, 18 and 19 (Taylor-Papadimitriou and Lane, 1987; Guelstein *et al*., 1988; Rudland and Hughes, 1989). Myoepithelial cells can be specifically stained with antibodies to smooth muscle actin (Bussolati *et al*., 1980) and to CD10 [anti-common acute lymphoblastic leukaemia antigen (anti-CALLA)] (Mahendran *et al*., 1989).

We used these markers for luminal and myoepithelial cells together with MAb MR6 to analyse the distribution of gp200-MR6 in normal and hyperplastic tissue samples and a panel of other markers to study the expression of gp200-MR6 in the hyperplasia cases. Our data indicate that gp200-MR6 is homogeneously expressed on both luminal and myoepithelial cells in the normal mammary duct, heterogeneously expressed in benign hyperplasia, and is down-regulated in invasive carcinomas of the breast.

### Materials and methods

### *Tissue samples*

Since the epitope detected by MAb MR6 cannot be detected in formalin-fixed paraffin-embedded tissue, fresh frozen tissue samples were used throughout this study. However, extensive attempts to retrieve the gp200-MR6, applying commonly used antigen retrieval methods such as enzymatic treatment, microwaving and pressure cooking were carried out. Samples from 75 breast tissues (14 normal, 21 *in situ* carcinoma, 32

Correspondence: MA Ritter
Received 30 November 1995; revised 28 March 1996; accepted 19 April 1996

invasive carcinoma and eight benign hyperplasia samples) were obtained from the Departments of Medical Oncology and Histopathology, Charing Cross Hospital, London, and from the Department of Histopathology, Royal Sussex County Hospital, Brighton. Paediatric thymus samples were obtained from children undergoing cardiac surgery at Great Ormond Street Hospital, London. Samples were snap-frozen in liquid nitrogen and stored at −70°C until sectioned.

*Antibodies*

Monoclonal antibody MR6 was produced as a culture supernatant in our laboratory (De Maagd *et al.*, 1985). Anti-E-cadherin (HECD-1) and anti-smooth muscle actin (sm-actin) antibodies were generous gifts from Dr M Pignatelli, Department of Histopathology, Royal Postgraduate Medical School (RPMS). HMFG1 antibody directed against a polymorphic epithelial mucin (MUC-1) was a gift from Professor A Epenetos, Department of Clinical Oncology, RPMS (Burchell *et al.*, 1983). Anti-cytokeratin (CK1-LP34), Dako-CD10 (anti-CALLA), Dako-Ki-67 antibodies and the secondary layer peroxidase-conjugated rabbit anti-mouse immunoglobulin were obtained from Dako (Copenhagen, Denmark). Anti-IL-4 receptor antibody was from Genzyme (West Malling, UK). All monoclonal antibodies used in this study were of $IgG_1$ isotype, except anti-sm-actin, which was $IgG_{2a}$.

*Antigen retrieval*

Different approaches were conducted on formalin-fixed, paraffin-embedded sections of normal and tumour breast tissues for retrieving the gp200-MR6 molecule. For comparison, fresh snap-frozen cryostat breast sections were immunostained in parallel. Anti-cytokeratin Dako-CK1 antibody was used as a positive control.

**Figure 1** Diagrammatic description of the ultrastructure of a normal mammary duct showing the relationship between different layers.

*Trypsin treatment* Dewaxed slides were incubated in a 0.1% trypsin solution (in 0.1% calcium chloride, pH 7.8) for 10 or 15 min at 37°C and then rinsed with phosphate-buffered saline (PBS).

*Microwave* Citrate buffer (0.01 M, pH 6.0) was preheated in a microwave for 2 min at 750 W. The slides were placed in the preheated citrate buffer and microwaved for 5, 10 or 15 min at 750 W. After treatment the slides were allowed to cool and were then rinsed in PBS (Shi *et al.*, 1991).

*Pressure cooking* Citrate buffer (0.01 M, pH 6.0) filling one-third of a domestic stainless-steel pressure cooker was brought to the boil on a hot plate. The dewaxed sections held in a stainless-steel rack were then lowered quickly into the boiling buffer and the lid tightly replaced. After approximately 5 min, when the cooker reached optimum pressure (103 kPa/1 5 p.s.i.) and the steam started to escape, the cooker was depressurised and cooled under running water. The sections were then cooled down in PBS, without being allowed to dry (Norton *et al.*, 1994).

*Immunohistochemical analysis*

Samples were analysed by the indirect immunoperoxidase technique using MAb MR6 and a panel of other antibodies (Table I). Frozen sections were cut at −30°C in a cryostat (Bright Instrument Company, UK) at 6 μm, mounted on poly-L-lysine-coated multispot slides (Hendley, Essex, UK), allowed to air dry for 2–24 h and then acetone fixed for 10 min. Sections were incubated with the primary antibodies for 1 h. MAb MR6 was used as a neat culture supernatant and the remaining antibodies were used at concentrations determined by previous titrations or as recommended by the manufacturers (Table I). Sections were then incubated with the secondary peroxidase-conjugated rabbit anti-mouse antibody, diluted 1:100 in PBS, for 30 min at room temperature. To prevent any possible cross-reactivity with endogenous Ig in the human tissue, 5% (v/v) normal human serum (NHS) was added to the secondary antibody preparation. Visualisation was achieved by a final incubation with diaminobenzidine tetrahydrochloride substrate (DAB; Sigma, UK) at 0.6 mg $ml^{-1}$, with 0.05% hydrogen peroxide added before use. The sections were then briefly counterstained with haematoxylin for 1 min and mounted in Kaiser's gelatin-based mountant. Irrelevant isotype-matched ($IgG_1$) antibody or omission of the primary antibody layer was used as a negative control. Anti-cytokeratin antibody (CK1-LP34), which reacts with human keratins, was used as positive control. Sections were scored for the cellular distribution of immunolabelling and for the intensity of

**Table I** Antibodies used in this study and their antigens, classes and titrations

| *Antibody*[a] | *Specificity* | *Ig Class* | *Dilutions* |
|---|---|---|---|
| MR6 | Gp200-MR6 | IgG1 | Neat |
| HECD-1 | E-cadherin | IgG1 | Neat |
| CK1-LP34 | Human keratins (6 and 18) | IgG1 | 1:50 |
| Dako-CD10 | CALLA | IgG1 | 1:50 |
| Anti-sm-actin | Smooth muscle actin | IgG2a | 1:1000 |
| Anti-IL-4R | IL-4 receptor | IgG1 | 1:10 |
| Dako-Ki-67 | Nuclear antigens | IgG1 | 1:50 |
| HMFG-1 | MUC-1 | IgG1 | 1:40 |
| Peroxidase-conjugated rabbit Ig | Mouse IgG | Polyclonal | 1:100 |

CALLA, common acute lymphoblastic leukaemia antigen; HECD-1, human epithelium cadherin-1; HMFG-1, human milk fat globule-1; MUC-1, mucin-1. [a]All antibodies used were mouse anti-human monoclonal antibodies except the secondary antibody, which was a polyclonal peroxidase-conjugated rabbit anti-mouse immunoglobulin.

labelling relative to the positive control used (− for no staining, + for weak, + + for moderate and + + + for strong staining). Although subjective, this method has been shown to give a high degree of concordance between independent observers (Luqmani *et al.*, 1989). Statistical analysis was performed using the chi-square test.

*SDS-PAGE and Western blotting*

Tissue lysates were derived from frozen sections of the same samples as those used for immunohistochemical analysis. Lysates were prepared from benign hyperplasia, carcinoma *in situ* and invasive carcinoma of the breast. Lysates from normal human thymuses, in which the gp200-MR6 is found in abundance, were used as a positive control. Ten sections (12 μm thick) were cut from each sample at −25°C and placed in prechilled Eppendorf microfuge tubes. Since each block was prepared to have approximately the same dimensions, this ensured that a similar amount of material was analysed for each tissue sample. Tubes were transferred to ice and 110 μl of lysis buffer [10 mM Tris/HCl, pH 7.2, 150 mM sodium chloride, 0.5% Nonidet P-40; (NP-40, Sigma)], containing 1 mM of protease inhibitor phenyl methyl sulphonyl fluoride (PMSF), was added to each tube. After 15 min, the lysates were centrifuged at 14 000 r.p.m. at 4°C for 5 min. The resulting cell-free supernatants were collected and mixed with equal volumes of double strength non-reducing Laemmli sample buffer [10% (w/v) sodium dodecyl sulphate (SDS), 10% (v/v) glycerol, 1 M Tris/HCl pH 6.8, 0.1% bromophenol blue] and boiled for 2–5 min at 100°C. The samples were allowed to cool for 5 min, aliquoted and either analysed immediately or stored at −20°C until required. The proteins in the lysates were separated using 7.5% SDS-PAGE (Laemmli, 1970) in a minigel apparatus (Hoefer Scientific Instruments, USA), and then transferred electrophoretically (300 mA for 60–90 min) onto a nylon membrane (Millipore, UK). Unoccupied charged sites on the membrane were blocked by immersion in 2.5% (w/v) skimmed milk powder (Marvel, Cadbury Schweppes, UK) in PBS for 24 h at 4°C. The nylon membrane was then incubated with MAb MR6 for 60 min with agitation at room temperature, washed in 0.5% (w/v) milk powder and then incubated with peroxidase-conjugated rabbit anti-mouse Ig [diluted 1:100 in 0.5% (w/v) milk powder] for 45 min with agitation at room temperature. Bands were visualised by incubating the membrane with DAB/0.05% hydrogen peroxide for 10 min, or alternatively, in order to get a higher sensitivity, by using the enhanced chemiluminescence method (ECL, Amersham, UK).

### Results

*Antigen retrieval*

Dewaxed, rehydrated, paraffin-embedded breast sections were treated with trypsin and then stained with MAb MR6 or anti-cytokeratin antibody. Snap-frozen fresh tissue sections were stained with MAb MR6 at the same time as a control for the effectiveness of the MR6 staining. Anti-cytokeratin antibody stained the epithelium in the paraffin-embedded breast tissue sections treated with trypsin, but it was not possible to obtain any MR6 staining. The frozen sections did, on the other hand, show a normal MR6 staining pattern. Similar results were also obtained when samples were treated with microwaving, pressure cooking or a combination of these methods. However, longer periods of exposure to microwave heat (10–15 min) led to massive tissue damage and subsequent lifting from the slides. The fact that this antigen cannot be detected in formalin-fixed tissue samples has made it difficult to collect a larger number of samples of benign hyperplasia of the breast, for this condition is usually diagnosed by chance in tissue resected for other reasons.

*Immunohistochemical analysis*

Screening of all breast tissues (normal, benign or malignant) was carried out by indirect immunoperoxidase technique and the results scored as: (−) for no staining at all, (+) for weak,

**Figure 2** Percentages of gp200-MR6-positive breast samples stained with MAb MR6 using immunoperoxidase method, (−) for no staining, (+) for weak, (+ +) for moderate, and (+ + +) for strong staining. (**a**) Normal. (**b**) *In situ*. (**c**) Invasive.

(+ +) for moderate and (+ + +) for strong staining. Anti-cytokeratin staining was scored as + + + since the intensity of staining was very strong in all samples studied. The staining intensity of the MAb MR6 was scored relative to that obtained with the anti-cytokeratin antibody and with the negative control. MAb MR6 was able to detect the gp200-MR6 on the ducts in all the normal breast tissue samples studied. The staining intensity was scored as + + in 86% of the normal samples, whereas in 7% of the normal samples the staining intensity was weaker (score +), and in another 7% the staining was very strong (score + + +) (Figure 2). The staining was homogeneous on luminal epithelium (Figure 3a, small arrowhead) and myoepithelium (Figure 3a, large arrowhead; 3b, LP34). In carcinoma *in situ* samples the expression of gp200-MR6 was less intense overall, with 67% of the samples scoring + and only 7% scoring + +. Interestingly, 27% of the cases were negative (−). In samples of invasive carcinoma the expression was completely lost in 71% of the cases, 18% were + and only 12% were + +. Chi-square analysis revealed that the proportion of positive–negative samples was significantly different ($P < 0.001$) in normal tissue, *in situ* carcinoma and invasive carcinoma (Table II).

Sections that contained both normal and tumour tissue made it possible to make a direct comparison between expression of the gp200-MR6 molecule in normal and transformed cells. Figure 3c shows strongly stained (+ +)

**Figure 3** Consecutive breast sections showing a normal mammary duct stained with MAb MR6 (**a**) and with LP34 (**b**). (**c**) MR6-positive normal ducts (small arrowheads) adjacent to an area of MR6-negative invasive carcinoma cells (large arrowhead). LP34 stains the normal and carcinoma cells equally (**d**). (**e**) A heterogeneous staining of a hyperplasia sample with MAb MR6, compared with the homogeneous staining with LP34 (**f**). Indirect immunoperoxidase staining; original magnification × 200.

normal ducts (small arrowheads) located in an area of MR6-negative invasive carcinoma cells (large arrowhead). LP34 stained both normal and abnormal cells equally (Figure 3d). In all the eight cases of benign hyperplasia of the breast studied, unusual heterogeneous expression of gp200-MR6 was observed (Figure 3e). This pattern was seen only in the hyperplastic inner layer of the duct (luminal epithelium) where some cells were MR6-positive and others were MR6 MR6-negative. These cells were not stained with antibodies to CD10 and to sm-actin, but they were strongly and homogeneously stained with LP34 (Figure 3f) and HMFG-1 (indicating that they were luminal epithelial cells). The outer layer (myoepithelium, CD10 positive, sm-actin positive) was homogeneously MR6-positive (Figure 4, large arrowhead). The MR6-positive luminal epithelial cells were bound together in a clumpy form and appeared to be linked to the basal layer (Figure 4, small arrowheads).

Consecutive sections of each tissue were stained with other antibodies, such as LP34 (Figure 3f), HECD-1 and anti-IL-4R. All these antibodies stained the hyperplastic ducts homogeneously, with no negative cells observed. Anti-smooth muscle actin and Dako-CD10 (anti-CALLA) antibodies clearly stained the myoepithelial layer, whereas no staining of the hyperplastic cells was observed. The Ki-67 antibody, which reacts with a nuclear antigen in proliferating cells during late $G_1$, S, M and $G_2$ phases of the cell cycle, but not in cells in the resting phase $G_0$ (Gerdes *et al.*, 1984), stained the majority of the MR6-positive cells, and also some of the MR6-negative cells.

Furthermore, some morphological differences were observed between the MR6-positive and the MR6-negative cells. The nuclei of MR6-positive cells were larger and rounder, whereas the nuclei of MR6-negative cells were relatively smaller and oval in shape (Figure 4).

**Table II** The proportion of gp200-MR6-expressing samples

| *Category* | *No. of samples* | *MR6-positive*[a] | *(%)* |
|---|---|---|---|
| Normal tissue | 14 | 14 | 100 |
| *In situ* carcinoma | 21 | 16 | 76 |
| Invasive carcinoma | 32 | 10 | 31 |

[a]Significantly different ($P < 0.001$), analysed by chi-square test.

**Figure 4** Frozen breast section of a hyperplasia sample stained with MAb MR6, showing the heterogeneous expression of gp200-MR6. The MR6-positive luminal epithelial cells are bound together in grape-like clumps, and appear to be linked to the basal layer (small arrowheads). Notice the morphological differences between the MR6-positive and the MR6-negative cells; the nuclei of MR6-positive cells are larger and rounder, whereas the nuclei of MR6-negative cells are relatively smaller and oval in shape. Indirect immunoperoxidase staining; original magnification ×400.

### *SDS-PAGE and Western blotting*

Lysates from normal breast tissue, benign hyperplasia, *in situ* carcinoma, or from invasive malignant tissues were run on SDS-PAGE, followed by transfer to a nylon membrane for Western blotting. The MAb MR6 detected a molecule of approximately 200 kDa (sometimes 210 kDa) in all the different lysates. The same 200 kDa band was detected in a lysate from a normal human thymus, which was used as a positive control (Figure 5). The intensity of the bands detected gave an indication of the amount of gp200-MR6 present in each sample. The normal thymic lysate (HT) contained the largest quantity of the gp200-MR6 molecule and invasive carcinoma (IC) of the breast contained the least. Other samples showed intermediate levels of gp200-MR6, with normal (NB) expressing levels greater than hyperplasia (BH), which in turn were greater than carcinoma *in situ* (IS). This observation is in agreement with the immunohistochemical data.

### Discussion

Previous studies on the expression of the IL-4R-associated gp200-MR6 molecule have indicated that, while all bladder tumours express the gp200-MR6 molecule, only approximately 30% of tumours of the breast, lung and colorectal tumours are MR6-positive (Al-Jabaari *et al.*, 1989; Mat *et al.*, 1990; Tungekar *et al.*, 1991,1996). In this paper we present a more detailed analysis of both benign and malignant tumours of the breast. Our data reveal that gp200-MR6 expression is associated with benign and most *in situ* tumours, while it is lost from the majority of invasive carcinomas, thus raising the possibility that loss of gp200-MR6 may play a role in tumorigenesis. Interestingly, we also observed heterogeneity of expression of gp200-MR6 within individual samples of benign hyperplasia; this correlates with morphological heterogeneity and may have implications for disease progression.

In breast cancer, gene amplification and/or overexpression of certain antigens (such as c-*erb*B-2, also known as HER-2), have been associated with metastasis and poor prognosis (Slamon *et al.*, 1987). The c-*erb*B-2 oncogene becomes pathologically activated by a truncation of its extracellular ligand-binding domain leading to continuous triggering of cell division (Downward *et al.*, 1984). Abnormal expression of such molecules can be due to either amplification or structural alteration of the normal cellular proto-oncogene encoding the receptor protein, resulting in either an increase in the number of receptor molecules per cell or in a structural variant of the receptor (Ullrich *et al.*, 1984). However, considerable attention in recent years has focused on the possible role of recessive oncogenes or tumour-suppressor

**Figure 5** Western blot analysis of tissue lysates from normal thymus (HT), normal breast (NB), benign hyperplasia (BH), *in situ* carcinoma (IS), and from invasive carcinoma samples (IC). Bands were visualised by using ECL method.

genes, whose activation, expression or introduction results in the suppression or inhibition of the tumorigenic phenotype. For example, the loss of tumour-suppressor gene *nm*23, which normally regulates development, has been observed to be associated with enhanced malignant potential (Steeg *et al.*, 1988; Bevilacqua *et al.*, 1989).

We have analysed the distribution of the gp200-MR6 in normal and transformed breast tissue in terms of intensity of expression and its distribution in different types of breast tissues. In normal breast tissues, gp200-MR6 is expressed on both luminal epithelium and myoepithelium (moderate in 86% of cases, strong in 7% of cases). In benign hyperplastic tissues, the expression of gp200-MR6 was heterogeneously distributed, with moderate staining (+ +) in some cells and no staining (−) in others. The gp200-MR6 was weak (+) in carcinoma *in situ* tissue samples and absent in the majority (71%) of invasive tumour samples.

These data raise the possibility that gp200-MR6 may act as the product of a tumour-suppressor gene. This molecule has previously been shown to be functionally associated with the receptor for IL-4 (Larché *et al.*, 1988; Imami *et al.*, 1994); its anti-tumour effect may therefore be mediated via the action of IL-4 which itself has been shown to have an anti-tumour action. Competitive binding of [$^{125}$I]IL-4 demonstrated the presence of 2000 high-affinity IL-4 binding sites per cell on the HT29 colorectal carcinoma cell line (Toi *et al.*, 1992), and human melanoma and ovarian carcinoma cell lines also express high-affinity IL-4R. Moreover, IL-4 has been shown to have an anti-proliferative effect on colorectal carcinoma cells *in vitro* (Tepper *et al.*, 1989) and an anti-tumour effect *in vivo* in the nude mouse xenograft model (Lahm *et al.*, 1994). The role, if any, of gp200-MR6 in such tumour suppression is currently under investigation. However, our observation that in hyperplasia of the breast the gp200-MR6 molecule is lost while IL-4R continues to be expressed is consistent with its proposed tumour-suppression function.

The immunolabelling of the epithelial hyperplasia samples showed an interesting differential labelling pattern with MR6-positive and MR6-negative cell population within a single tissue. The strange pattern of MR6-positive cells, which was arranged in grape-like clumps (Figure 4), raised the question of whether these cells (MR6-positive) originated from the myoepithelial layer. However, antibodies that are used routinely to delineate myoepithelium (anti-smooth muscle actin, CALLA) did not stain these MR6-positive clumps, instead they defined clearly the myoepithelial and basement membrane layers, indicating that the stained hyperplastic cells in the lumen were not myoepithelial. Furthermore, HMFG-1 gave a strong and homogeneous staining of the cells in question confirming that they were luminal. Carcinoma *in situ*, a proliferation of presumably malignant cells, confined to the mammary ducts with no evidence of invasion through the basement membrane into the surrounding stroma, is sometimes mistaken for atypical hyperplasia, which is a non-malignant proliferation of epithelial cells. The histological differentiation of pure ductal carcinoma *in situ* (DCIS) from atypical ductal hyperplasia (ADH) is usually difficult, and some cases may create diagnostic disagreement among histopathologists. It has been found that ADH has a significant role as a marker for high risk of breast cancer development. Moderate or severe benign hyperplasia, where cells show a mild variation of cytological pattern with both oval and round nuclei, were found to have a slight elevation of breast cancer risk (Page *et al.*, 1985; Tavassoli and Norris, 1990), and in a recent study it has been found that 17% of DCIS cases were associated with ADH (Lennington *et al.*, 1994). In this study we have analysed eight cases of moderate hyperplasia (five to ten epithelial layers), and two of them were associated with DCIS components. The heterogeneity in gp200-MR6 expression that we have observed in hyperplasia, and the variation in cell morphology, may be helpful as a prognostic measure, where the increased variation in gp200-MR6 expression and in cytological pattern may indicate an increased risk of malignant transformation.

In conclusion, we have shown that, while gp200-MR6 is expressed on normal epithelial cells, benign tissue and *in situ* tumours, it is lost from the invasive carcinomas of the breast. Moreover, its expression within individual hyperplastic samples is heterogeneous. Analysis of this molecule may therefore be useful in tumour grading and prognostic evaluation. Further, functional studies are required to test the hypothesis that gp200-MR6 has tumour-suppressive function and that its loss is a primary cause of tumorigenesis.

**Acknowledgements**

We would like to thank Dr M Pignatelli and Ms S Van Noorden for their valuable discussions. We would also like to thank Ms R Coope and Mr D Peston for their help during tissue sectioning, and antigen retrieving experiments. This research was supported by the Cancer Research Campaign and the Secretariat of Higher Education of Libya (AAA).

### References

AL-JABAARI B, LADYMAN HM, LARCHÉ M, SIVOLAPENKO GB, EPPENETOS AA AND RITTER MA. (1989). Elevated expression of the Interleukin 4 receptor in carcinoma: a target for immunotherapy? *Br. J. Cancer*, **59**, 910–914.

BEVILACQUA G, SOBEL ME, LIOTTA LA AND STEEG PS. (1989). Association of low nm23 RNA levels in human primary infiltrating ductal breast carcinomas with lymph node involvement and other histopathological indicators of high metastatic potential. *Cancer Res.*, **49**, 5185–5190.

BURCHELL J, DURBIN H AND TAYLOR-PAPADIMITRIOU J. (1983). Complexity of expression of antigenic determinants, recognized by monoclonal antibodies HMFG-1 and HMFG-2, in normal and malignant human mammary epithelial cells. *J. Immunol.*, **131**, 508–513.

BUSSOLATI G, BOTTA G AND GULIOTTA P. (1980). Actin-rich (myoepithelial) cells in ductal carcinoma *in situ* of the breast. *Virchows Arch.* (B), **34**, 251–259.

DE MAAGD R, MACKENZIE W, SCHUURMAN H, RITTER MA, PRICE K, BROEKHUIZEN R AND KATER L. (1985). The human thymus microenvironment: heterogeneity detected by monoclonal anti-epithelial cell antibodies. *Immunology*, **54**, 745–754.

DOWNWARD, J, YARDEN Y, MAYES E, SCRACE G, TOTTY N, STOCKWELL P, ULLRICH A, SCHLESSINGER J AND WATERFIELD M. (1984). Close similarity of epidermal growth factor receptor and *erb*B-2 oncogene protein sequences. *Nature*, **307**, 521–527.

GERDES J, LEMKE H, BAISCH H, WACKER H, SCHWAB U AND STEIN H. (1984). Cell cycle analysis of a cell proliferation-associated human nuclear antigen defined by the monoclonal antibody Ki-67. *J. Immunol.*, **133**, 1710–1715.

GUELSTEIN V, TCHYPYSHEVA T, ERMILOVA V, LITVINOVA, L, TROYANOVSKY S AND BANNIKOV G. (1988). Monoclonal antibody mapping of keratins 8 and 17 and of vimentin in normal human mammary gland, benign tumours, dysplasias and breast cancer. *Int. J. Cancer*, **42**, 147–153.

IMAMI N, LARCHÉ M AND RITTER MA. (1994) Inhibition of alloreactivity by mAb MR6: differential effect on IL-2- and IL-4-producing human T cells. *Int. Immunol.*, **6**, 1575–1584.

LAEMMLI UK. (1970). Cleavage of structural proteins during the assembly of the head of the bacteriophage T4. *Nature*, **227**, 680–685.

LAHM H, SCHNYDER B, WYNIGER J, BORBENYI Z, YILMAZ A, CAR B, FISHCHER J, GIVEL J AND RYFFEL B. (1994). Growth inhibition of human colorectal-carcinoma cells by interleukin-4 and expression of functional interleukin-4 receptors. *Int. J. Cancer*, **59**, 440–447.

LARCHÉ M, LADYMAN H AND RITTER MA. (1987). Human thymic epithelial cells and lymphocytes may share common epitopes. In *Leukocyte Typing III*, McMichael A, Beverly N, Hogg N and Horton M., (eds) pp. 257–259. *Oxford University Press: Oxford.*

LARCHÉ M, LAMB J, O'HEHIR R, IMAMI N, ZANDERS E, QUINT D, MOQBEL R AND RITTER MA. (1988). Functional evidence for a monoclonal antibody that binds to the human IL-4 receptor. *Immunology*, **65**, 617–622.

LENNINGTON W, JENSEN R, DALTON L AND PAGE D. (1994). Ductal carcinoma *in situ* of the breast. Heterogeneity of individual lesions. *Cancer*, **73**, 118–124.

LUQMANI YA, BENNETT C, PATERSON I, CORBISHLEY C, RIO M, CHAMBON P AND RYALL G. (1989). Expression of the pS2 gene in normal, benign and neoplastic human stomach. *Int. J. Cancer*, **44**, 806–812.

MAHENDRAN R, MCILHINNEY R, O'HARE M, MONAGHAN P AND GUSTERSON B. (1989). Expression of the common acute lymphoblastic leukaemia antigen (CALLA) in the human breast. *Mol. Cell. Probes*, **3**, 39–44.

MAT I, LARCHÉ M, MELCHER D AND RITTER MA. (1990). Tumour-associated upregulation of IL-4 receptor complex. *Br. J. Cancer*, **62**, (suppl. x), 96–98.

NORTON A, JORDAN S AND YEOMANS P. (1994). Brief, high-temperature heat denaturation (pressure cooking): a simple and effective method of antigen retrieval for routinely processed tissue. *J. Pathol.*, **173**, 371–379.

PAGE DL, DUPONT WD, ROGERS LW AND RADOS MS. (1985). Atypical hyperplasia lesions of the female breast. *Cancer*, **55**, 2698–2708.

RUDLAND P AND HUGHES C. (1989). Immunocytochemical identification of cell types in human mammary gland: variations in cellular markers are dependent on glandular topography and differentiation. *J. Histochem. Cytochem.*, **37**, 1087–1100.

SHI SR, KEY ME AND KALRA KL. (1991). Antigen retrievel in formalin-fixed, paraffin-embedded tissues: an enhancement method for immuno-histochemical staining based on microwave oven heating of tissue sections. *J. Histochem. Cytochem.*, **39**, 741–748.

SLAMON DL, CLARKE GM, WONG SG, LEVIN WJ, ULLRICH A AND MCGUIRE WL. (1987). Human breast cancer: correlation of relapse and survival with amplification of the HER-2/*neu* oncogene. *Science*, **235**, 177–182.

STEEG P, BEVILACQUA G, POZZATTI R, LIOTTA L AND SOBEL M. (1988). Altered expression of *NM23*, a gene associated with low tumour metastasis potential, during adenovirus 2 Ela inhibition of experimental metastasis. *Cancer Res.*, **48**, 6550–6554.

TAVASSOI FA AND NORRIS HJ. (1990). A comparison of the results of long-term follow-up for atypical introductal hyperplasia and intraductal hyperplasia of the breast. *Cancer*, **65**, 518–529.

TAYLOR-PAPADIMITRIOU J AND LANE E. (1987). Keratin expression in the mammary gland. In *The Mammary Gland: Development, Regulation and Function*, Neville M and Daniel C (eds) pp. 181–215. Plenum: New York.

TEPPER R, PATTENGALE P AND LEDER P. (1989). Murine interleukin-4 displays potent anti-tumour activity *in vivo*. *Cell*, **57**, 503–512.

TOI M, BICKNELL R AND HARRIS AL. (1992). Inhibition of colon and breast carcinoma cell growth by interleukin-4. *Cancer Res.*, **52**, 275–279.

TUNGEKAR MF, TURLEY H, DUNNILL MS, GATTER KC, RITTER MA AND HARRIS AL. (1991) Interleukin-4 receptor expression on human lung tumours and normal lung. *Cancer Res.*, **51**, 261–264.

TUNGEKAR MF, GATTER KC AND RITTER MA. (1996). Bladder carcinomas and normal urothelium universally express gp200-MR6, a molecule functionally associated with the interleukin-4 receptor (CD124). *Br. J. Cancer*, **73**, 429–432.

ULLRICH A, COUSSENS L, HAYFLICK JS, DULL TJ, GRAY A, TAM AW, LEE J, YARDEN Y, LIBERMANN T AND SCHLESSINGER J. (1984). Human epidermal growth factor receptor cDNA sequence and aberrant expression of the amplified gene in A431 epidermoid carcinoma cells. *Nature*, **309**, 418–425.

VON GAUDECKER B, KENDALL M AND RITTER MA. (1996). Immuno-electron microscopy of the thymic microenvironment. *Microscopy Res. Tech.*, (in press).